# Hybrid sequence-based analysis reveals the distribution of bacterial species and genes in the oral microbiome at a high resolution

**DOI:** 10.1016/j.bbrep.2024.101717

**Published:** 2024-04-26

**Authors:** Masaya Yamaguchi, Toshihiro Uchihashi, Shigetada Kawabata

**Affiliations:** a Bioinformatics Research Unit, Osaka University Graduate School of Dentistry, Suita, Osaka, Japan; b Department of Microbiology, Osaka University Graduate School of Dentistry, Suita, Osaka, Japan; c Bioinformatics Center, Research Institute for Microbial Diseases, Osaka University, Japan; d Center for Infectious Diseases Education and Research, Osaka University, Japan; e Department of Oral and Maxillofacial Surgery, Osaka University Graduate School of Dentistry, Suita, Osaka, Japan

**Keywords:** Oral microbiome, Bacterial single-cell sequencing, 16S rRNA sequencing, Metagenome shotgun sequencing

## Abstract

Bacteria in the oral microbiome are poorly identified owing to the lack of established culture methods for them. Thus, this study aimed to use culture-free analysis techniques, including bacterial single-cell genome sequencing, to identify bacterial species and investigate gene distribution in saliva. Saliva samples from the same individual were classified as inactivated or viable and then analyzed using 16S rRNA sequencing, metagenomic shotgun sequencing, and bacterial single-cell sequencing. The results of 16S rRNA sequencing revealed similar microbiota structures in both samples, with *Streptococcus* being the predominant genus. Metagenomic shotgun sequencing showed that approximately 80 % of the DNA in the samples was of non-bacterial origin, whereas single-cell sequencing showed an average contamination rate of 10.4 % per genome. Single-cell sequencing also yielded genome sequences for 43 out of 48 wells for the inactivated samples and 45 out of 48 wells for the viable samples. With respect to resistance genes, four out of 88 isolates carried *cfxA*, which encodes a β-lactamase, and four isolates carried erythromycin resistance genes. Tetracycline resistance genes were found in nine bacteria. Metagenomic shotgun sequencing provided complete sequences of *cfxA*, *ermF*, and *ermX*, whereas other resistance genes, such as *tetQ* and *tetM*, were detected as fragments. In addition, virulence factors from *Streptococcus pneumoniae* were the most common, with 13 genes detected. Our average nucleotide identity analysis also suggested five single-cell-isolated bacteria as potential novel species. These data would contribute to expanding the oral microbiome data resource.

## Highlights

• Bacterial single-cell seq of saliva suggested five potential novel species.
• A healthy oral microbiome contained AMR and virulence factor genes.
• Our data contribute to expanding oral microbiome data resource.

## 1 Introduction

The oral microbiome is the collection of microorganisms that inhabit the oral cavity and are present in the human saliva. The oral microbiome is influenced by various factors, including diet, oral hygiene, age, and health status [1]. In general, a healthy oral microbiome is dominated by bacteria belonging to the phyla Firmicutes, Actinobacteria, and Proteobacteria, including *Streptococcus*, *Rothia*, and *Neisseria* [1,2]. The oral microbiome plays an important role in maintaining oral and general health. Some bacteria in the oral microbiome produce antimicrobial compounds that prevent the growth of harmful bacteria in the oral cavity [3]. Oral microbiome dysbiosis is associated with systemic health conditions, such as cardiovascular diseases, diabetes, and respiratory infections [1,2].

Microbiome analyses have provided additional information regarding the structure of the oral microbiota. However, many unculturable bacteria are present, and the individual bacteria or genes in the oral microbiota remain unidentified [2]. Similar to the gut, the oral cavity may facilitate the accumulation of diverse bacteria and cross-species transfer of genes. Some novel capsular types of *Streptococcus pneumoniae* and drug resistance genes are derived from oral *Streptococcus* [[4], [5], [6], [7]]. In addition to cross-species gene transfer, mobile genetic elements, such as plasmids containing antimicrobial resistance (AMR) genes, are transmissible between bacteria. Thus, droplet infection of saliva could be a global source of drug resistance.

Amplicon and metagenomic shotgun sequencing have been used in microbiome analysis. Specifically, 16S rRNA amplicon sequencing has been used to analyze the structure of the microbiome, but its accuracy is insufficient to estimate phylogeny at the species level [8,9]. Metagenomic shotgun sequencing allows for the comprehensive analysis of gene structure. However, reconstructing reads in individual bacterial genomes via *de novo* assembly and binning is challenging [10]. Additionally, in-depth analysis requires a large number of reads, which increases the costs of sequencing and computational resources. Moreover, not all reads obtained can be used to analyze the bacterial layer owing to contamination of the host DNA.

Bacterial single-cell sequencing has also been used for microbiome analysis [11,12]. However, bacteria are approximately 1/10th the size and 1/1000th the amount of DNA in host cells [[13], [14], [15]]. A previous study that used single-cell sequencing only achieved an average genome completeness of approximately 14 % in 180 single bacterial cells [16]. Using single-amplified genome (SAG) gel technology, Hosokawa et al. obtained an average genome completeness of 31.8 % in 346 isolates [11].

Considering the lack of culture methods for identifying bacterial species in the oral microbiome, we aimed to use amplicon, metagenomic shotgun, and single-cell sequencing to identify bacterial species and investigate gene distribution in saliva samples. The bacterial genomes obtained showed a distribution of virulence factors and resistance genes with high accuracy and indicated the possibility of a novel genus.

## 2 Material and methods

### 2.1 Sample preparation

After fasting for >30 min in the morning, a healthy donor provided 5 mL of saliva. A 1 mL aliquot of the sample was added to OMNIgene ORAL solution (DNA Genotek Inc., Canada), which inactivates but stabilizes bacterial cells, and then stored at room temperature until single-cell sequencing. The presence of live bacteria was investigated as follows. In brief, 3 mL of saliva was centrifuged at 8000 rpm for 10 min, suspended in 800 μL of 50 % glycerol/RPMI1640 solution, and then stored at −30 °C until single-cell sequencing. The 16S rRNA sequencing, metagenome shotgun sequencing, single-cell isolation, genome amplification, and paired-end genome sequencing of both saliva samples were performed by bitBiome Inc. in Japan.

### 2.2 16S rRNA gene sequencing and analysis

The V3–V4 hypervariable regions of 16S rRNA genes were analyzed using the Illumina protocol for the preparation of 16S Metagenomic Sequencing Library. Barcoded amplicons, amplified with 341F and 806R primers (forward, 5′-TCGTCGGCAGCGTCAGATGTGTATAAGAGACAGCCTACGGGNGGCWGCAG-3′; reverse, 5′-GTCTCGTGGGCTCGGAGATGTGTATAAGAGACAGGACTACHVGGGTATCTAATCC-3′) were sequenced using the Illumina MiSeq 2 × 300 bp platform. The FASTQ data were analyzed by BitBiome Inc. Raw reads were processed with QIIME2 v.2020.2 [17] using DADA2 denoising paired with the options --p-trim-left-f 20, --p-trim-left-r 5, --*p*-trunc-len-f 0, and --*p*-trunc-len-r 250 [18]. After quality filtering, bacterial taxa were estimated using the feature-classifier classify-sklearn and a SILVA reference database [19]. The data were visualized using “taxa collapse” and “taxa barplot.”

### 2.3 Metagenome shotgun sequencing and analysis

Metagenome shotgun sequencing was performed as previously described [20]. Briefly, total DNA was extracted from the saliva samples by using The QIAamp PowerFecal Pro Kit (QIAGEN, Germany). Each sample was sequenced using Illumina MiSeq 2 × 75 bp. FASTQ data were analyzed by BitBiome Inc. The sequence reads were filtered using bbduk. sh v38.79 (https://sourceforge.net/projects/bbmap/) with the parameters qtrim = r, trimq = 10, minlength = 40, maxns = 1, and minavgquality = 15, and human genome contaminations were removed by mapping with bbmap. sh 38.79 with the parameters quickmatch fast untrim minid = 0.95, maxindel = 3, bwr = 0.16, bw = 12, minhits = 2, and human_masked_index qtrim rl trimq = 10 [21]. The processed sequences were assembled with Megahit v1.1.3 using default settings [22]. Metagenome-assembled genomes (MAGs) were constructed by binning with MetaBAT2 in metaWRAP v.1.3.2 using default settings [23,24]. The coding sequences of the contigs and MAGs were examined with Prokka v1.14.6 using the options --rawproduct and --mincontiglen 200 [25]. The number, total length, and GC content of the assembled contigs and MAGs were evaluated using QUAST v5.0.2 [26]. The assembled contigs were taxonomically classified with MetaPhlAn3 using default settings and mpa_v30_CHOCOPhlAn_201,901 [27], whereas the MAGs were taxonomically classified using GTDB-Tk v1.3.0 and database release 95 [28]. The completeness and contamination of the MAGs were calculated using CheckM v1.1.2 [29].

### 2.4 Bacterial single-cell genome sequencing with SAG-gel

Bacterial single-cell sequencings were performed as previously described [11]. Briefly, single cells were isolated into gel beads, and their genomes were amplified using the SAG gel method [11]. Single-gel beads harboring a SAG were sorted into individual wells of a 96-well microplate. Each SAG was sequenced using Illumina MiSeq 2 × 75 bp. The FASTQ data were analyzed by bitBiome Inc. Sequence reads were filtered using bbduk. sh v38.79, and human genome contaminations were removed by mapping with bbmap. sh 38.79 and metagenome shotgun sequencing. The processed sequences were assembled with SPAdes v3.14.0, using the settings --sc, --careful, --disable-rr, and --disable-gzip-output, and contigs >1000 bp were retained for subsequent analyses. The coding sequences were examined with Prokka v1.14.6 using the options --raw product and --mincontiglen 200 [25]. The contig number, total length, and GC content of the assembled genomes were evaluated using QUAST v5.0.2 [26]. Completeness and contamination were calculated using CheckM v1.1.2 [29]. The assembled genomes were taxonomically classified using GTDB-Tk v1.1.1 and the database release 95 [28]. The average nucleotide identity (ANI) values of the selected SAGs were calculated using FastANI 1.34 [30]. In this study, SAGs with ANI value > 99.9 were considered redundant [31].

### 2.5 Sequencing-based profiling

Sequencing-based profiling was performed as previously described [32]. Quality control and preprocessing of the FASTQ files from the next-generation sequencing were performed using fastp v.0.20.0 [33]. To identify the bacterial species, we performed an ANI analysis of the assemblies by using Microbial Genomes Atlas MiGA online (http://microbial-genomes.org/) [34]. The AMR and virulence factor profiles were determined using ARIBA 2.14.4, with cleaned sequencing data [35]. We used the Comprehensive Antibiotic Resistance Database (CARD) v.3.0.8 [36] and core and full datasets of the virulence factor database (VFDB) [37] as reference for AMR and virulence factor profiling, respectively. The minimum percentage identities for the assemblies were set to 93 and 90 for the CARD and other databases, respectively. The analyzed data were visualized using Phandango [38].

### Ethical statement

The study was conducted with written informed consent from the donor and approved by the Institutional Review Board of Osaka University Graduate School of Dentistry (R4-E4).

## 3 Results

### 3.1 Comparison of 16S rRNA, metagenome shotgun, and bacterial single-cell sequencing on the human salivary microbiome

**Fig. 1:**
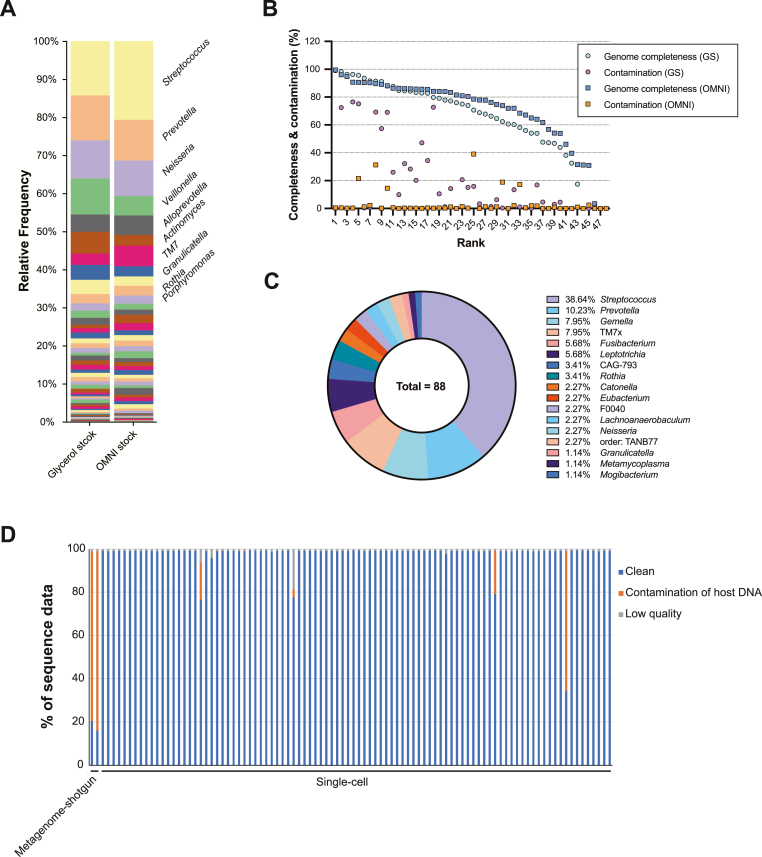
**Human saliva microbiome analysis using 16S rRNA, meta-genome shotgun, and bacterial single cell sequencing.** After fasting for more than 30 min in the morning, 5 mL of saliva was collected from a healthy donor. One mL was added to OMNIgene ORAL solution and then stored at room temperature. Another 3 mL of saliva was suspended in 800 μL of 50 % glycerol/RPMI1640 solution and then stored at −30 °C. (**A**) **Taxonomic bar plots by 16S rRNA analysis.** Individual taxonomy legend and relative frequency value are shown in Table S1. **(B) Genome completeness and contamination plots of single-cell analysis.** In 48 wells, single cell isolation, genome amplification, and paired-end genome sequencing of both saliva samples were performed by bitBiome. **(C) Taxonomy in single-cell analysis.** Combined results of SAGs prepared from glycerol and OMNI stocks are shown. Eight samples were excluded because those sequencing data were insufficient to identify the taxonomy. (**D**) **Human DNA contamination rate in metagenome shotgun and single-cell analyses.** Two samples in single-cell analyses were excluded because any sequencing data were obtained from the samples.

The workflow of the 16S rRNA sequencing, metagenome shotgun sequencing, 48-well single-cell isolation, and short-read genome sequencing of the inactivated and viable samples are shown in Fig. S1. Taxonomic bar plots at the genus level based on 16S rRNA analysis revealed that the inactivated and viable samples had similar microbiome structures. In both samples, *Streptococcus* was the predominant genus, followed by *Prevotera*, *Neisseria*, and *Veillonella* (Fig. 1A and Table S1). For bacterial single-cell analysis, genome sequences were obtained from 43 out of 48 wells for the OMNIgene-preserved samples and from 45 out of 48 wells for the glycerol stock samples. FastANI analysis revealed two redundant SAGs in glycerol stocks, OSU001s, and four redundant SAGs in OMNI stocks, OSU002s (Table S2). Genomic completeness greater than 80 % was achieved in 17 wells for the OMNIgene-preserved samples and in 24 wells for the glycerol stock samples compared with known genomic sequences (Fig. 1B and Table S3). Similar to the 16S rRNA sequencing results, single-cell sequencing results showed that *Streptococcus* was the most abundant genus in the samples, followed by *Prevotella*. By contrast, the percentage of *Neisseria*, which was high in the 16S rRNA sequencing, was low, and *Veillonella* and *Alloprevotella* were not detected. These differences in trends may be due to random bias caused by the extraction of some bacteria from the oral microbiome. Alternatively, they may reflect technical influences, such as the ease of single-cell isolation of each bacterium or differences in genome extraction rates between gram-positive and gram-negative bacteria in the 16S rRNA analysis. In addition, 60 bacterial genera were detected using 16S rRNA sequencing, whereas only 17 genera were detected using single-cell sequencing (Fig. 1A and C, and Table S3). While 16S rRNA sequencing is a targeted approach focusing on a specific gene, single-cell sequencing dissociates and sequences individual bacterial cell genomes. Therefore, a large number of SAGs must be obtained to identify the microbiome structure using single-cell analysis.

The total raw read counts for the metagenomic shotgun and single-cell analyses were 61,126,868 and 55,918,930, respectively (Tables S3 and S4). Metagenomic shotgun sequencing revealed an average contamination rate of 81.6 %, indicating the difficulty in separating bacterial DNA from human saliva-derived specimens (Fig. 1D and Table S4). By contrast, bacterial single-cell sequencing obtained a much lower average contamination rate of 10.4 % per genome because of the single-cell separation process (Fig. 1D and Table S3). Metagenome binning yielded nine bins from metagenome assemblies, of which eight were identified by metagenome shotgun sequencing and GTDBtk analysis, and 44 strains were identified at the species level by bacterial single-cell sequencing and GTDBtk analyses (Table S3 and Table 1).

**Table 1:** Summary of metagenome binning.

mag_id	comp.lwf	cont.lwf	n.contigs	largest_contig	total_length	gc	n.CDS	gtdbtk.taxonomy
OSU001-metabat2-bin.1	0	0	156	4400	331,827	63.33	322	–
OSU001-metabat2-bin.2	89.79	11.89	441	40,578	2319340	65.06	2066	d__Bacteria; p__Actinobacteriota; c__Actinomycetia; o__Actinomycetales; f__Actinomycetaceae; g__Pauljensenia; s__Pauljensenia sp000278725
OSU001-metabat2-bin.3	77.59	0.86	400	37,652	1634688	50.23	1742	d__Bacteria; p__Proteobacteria; c__Gammaproteobacteria; o__Burkholderiales; f__Neisseriaceae; g__Neisseria; s__Neisseria sp000186165
OSU001-metabat2-bin.4	51.81	1.7	686	15,076	1861607	41.77	1420	d__Bacteria; p__Bacteroidota; c__Bacteroidia; o__Bacteroidales; f__Bacteroidaceae; g__Prevotella; s__Prevotella jejuni
OSU001-metabat2-bin.5	70.45	39.91	723	18,835	1948412	40.51	1798	d__Bacteria; p__Firmicutes; c__Bacilli; o__Lactobacillales; f__Streptococcaceae; g__Streptococcus; s__Streptococcus salivarius
OSU002-metabat2-bin.1	68.97	0.86	427	26,905	1517593	50.11	1619	d__Bacteria; p__Proteobacteria; c__Gammaproteobacteria; o__Burkholderiales; f__Neisseriaceae; g__Neisseria; s__Neisseria sp000186165
OSU002-metabat2-bin.2	44.44	6.69	305	24,665	812,076	39.67	737	d__Bacteria; p__Firmicutes; c__Bacilli; o__Lactobacillales; f__Streptococcaceae; g__Streptococcus; s__Streptococcus salivarius
OSU002-metabat2-bin.3	79.36	5.3	579	23,070	2001365	64.98	1842	d__Bacteria; p__Actinobacteriota; c__Actinomycetia; o__Actinomycetales; f__Actinomycetaceae; g__Pauljensenia; s__Pauljensenia sp000278725
OSU002-metabat2-bin.4	57.45	1.12	148	7461	419,386	43.6	415	d__Bacteria; p__Patescibacteria; c__Saccharimonadia; o__Saccharimonadales; f__Saccharimonadaceae; g__TM7x; s__

comp.lwf and cont.lwf refer to the completeness and contamination calculated by the CheckM lineage workflow, respectively. n.contigs, total_length, gc, and n.CDS are the number of contigs, total number of nucleotide base pairs, GC content, and number of coding sequences, respectively. gtdbtk.taxonomy shows the phylogenetic classifications by GTDBTk.

### 3.2 Detection of antimicrobial resistance genes and virulence factor genes from metagenome shotgun and bacterial single-cell sequencing

**Fig. 2:**
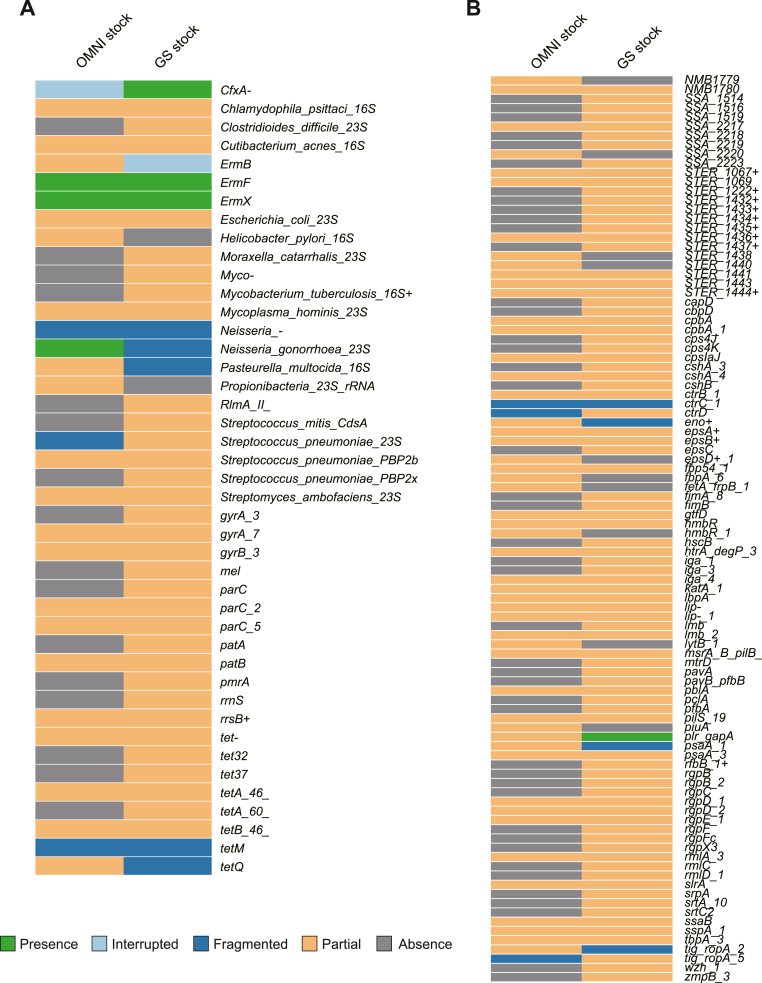
**(A) Burden of antimicrobial resistance (AMR) genes in the metagenome shotgun-sequenced samples.** Reference data were obtained from the Comprehensive Antibiotic Resistance Database (CARD). Green, light blue, blue, orange, and grey indicate matches to the reference, interrupted, fragmented, partial, and lacking genes, respectively. “Interrupted” means that all of reference genes were not represented in the assembly, “fragmented” means that the gene was assembled to ≥2 contigs, and “partial” means that the gene was not a complete one containing start and stop codons. (**B**) **Burden of genes encoding virulence factors in the metagenome shotgun-sequenced samples.** Reference data were obtained from Virulence Factor Database (VFDB) full dataset. Green, light blue, blue, orange, and grey indicate matches to the reference, interrupted, fragmented, partial, and lacked genes, respectively. The clustering trees were generated by ARIBA based on the gene distribution. Graphical data were obtained using Phandango. (For interpretation of the references to colour in this figure legend, the reader is referred to the Web version of this article.)

**Fig. 3:**
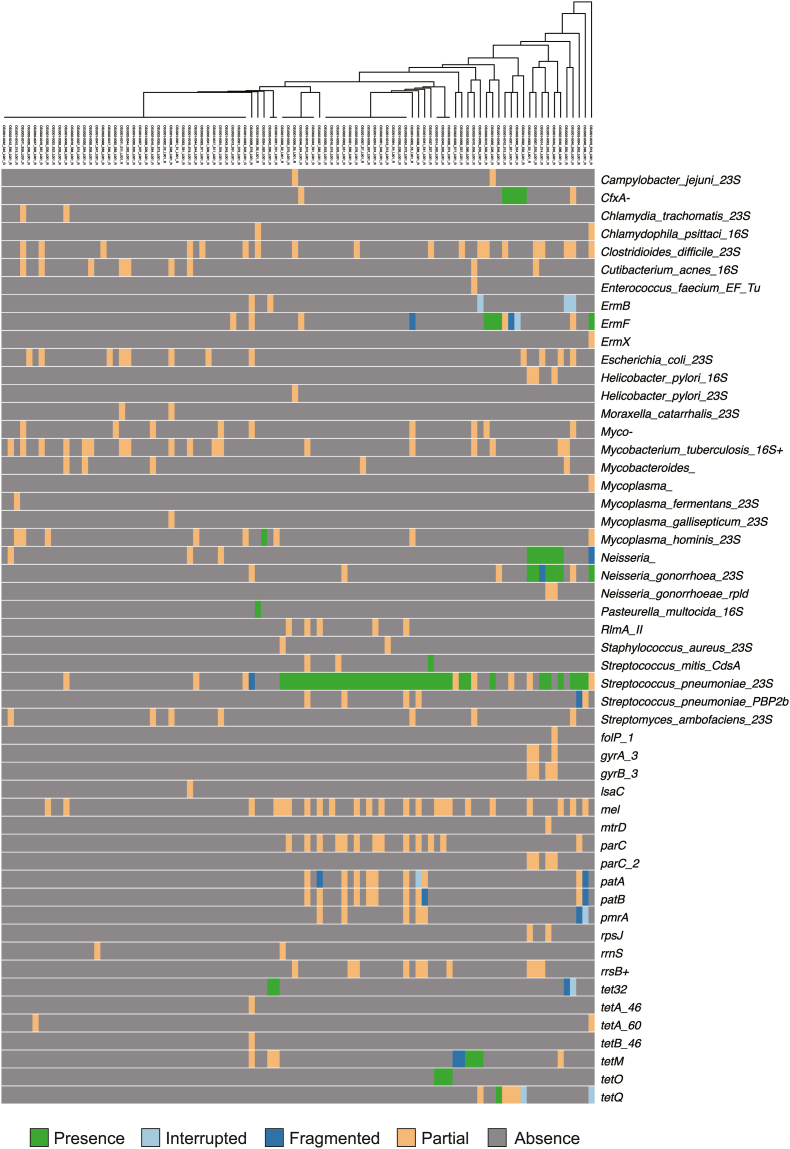
**Burden of AMR genes in the single-cell isolated bacteria.** Reference data were obtained from the Comprehensive Antibiotic Resistance Database (CARD). Green, light blue, blue, orange, and grey indicate matches to the reference, interrupted, fragmented, partial, and lacking genes, respectively. The clustering tree was generated by ARIBA based on the gene distribution. Graphical data were obtained using Phandango. (For interpretation of the references to colour in this figure legend, the reader is referred to the Web version of this article.)

To explore AMR gene distribution, we analyzed sequencing data using the program ARIBA 2.14.4 and AMR gene reference dataset CARD v3.0.8 [35,36]. Metagenome shotgun sequencing revealed that *cfxA* encoding a β-lactamase and the erythromycin resistance genes *ermF* and *ermX* were complete sequences, whereas the other AMR genes were fragmented (Fig. 2). Single-cell sequencing showed that four of the 88 isolates harbored *cfxA* and four harbored *ermF*. For tetracycline resistance, nine isolates carried *tet32*, *tetM*, *tetO*, or *tetQ* (Fig. 3).

**Fig. 4:**
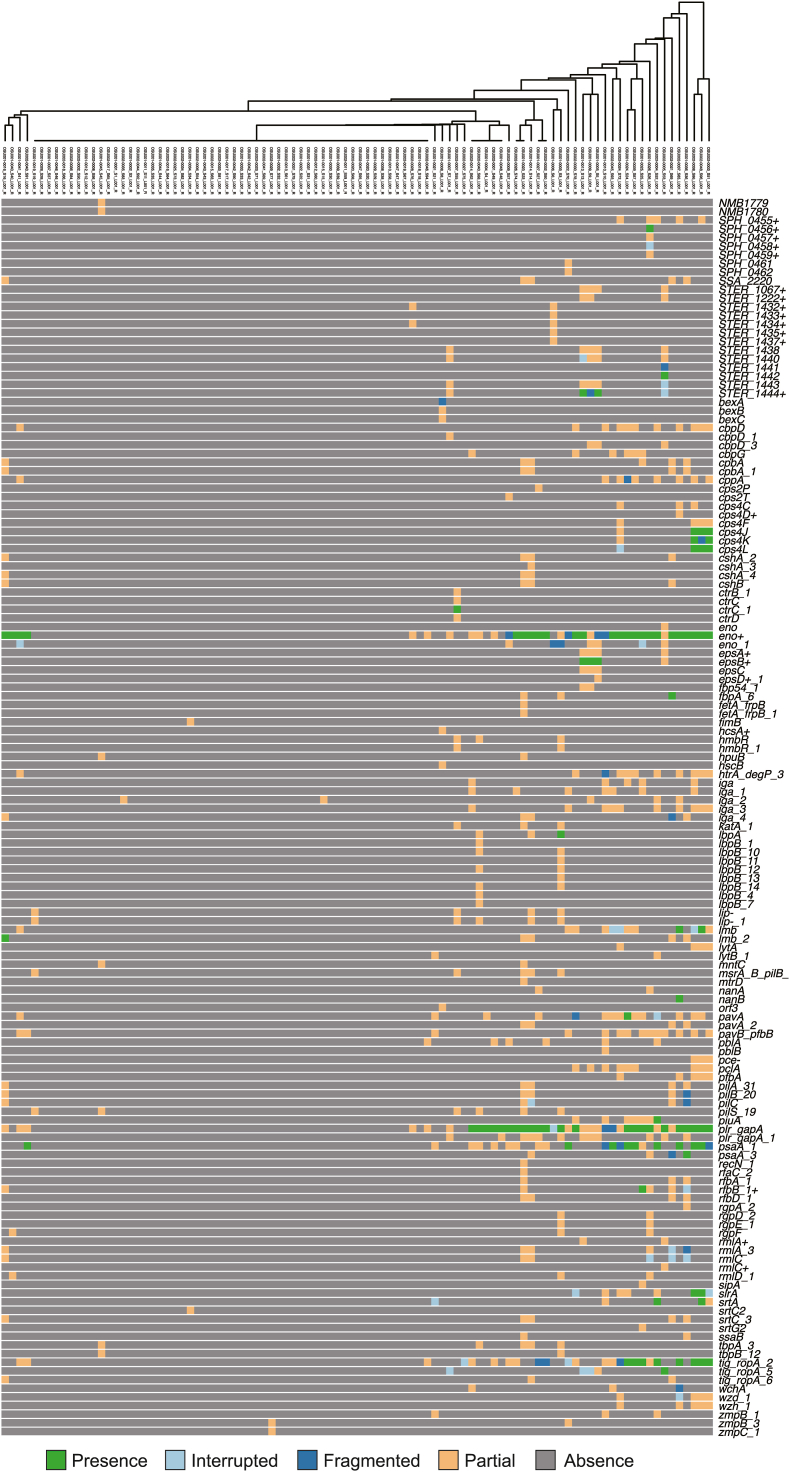
**Burden of genes encoding virulence factors in the single-cell isolated bacteria.** Reference data were obtained from the VFDB full dataset. Green, light blue, blue, orange, and grey indicate matches to the reference, interrupted, fragmented, partial, and lacked genes, respectively. The clustering tree was generated using ARIBA based on the gene distribution. Graphical data were obtained using Phandango. (For interpretation of the references to colour in this figure legend, the reader is referred to the Web version of this article.)

Both analyses detected fewer genes encoding virulence factors than AMR genes. Metagenome shotgun sequencing revealed intact *gapA*, an essential gene encoding an enzyme for glycolysis, whose ortholog inhibits the biological effects of C5a on human neutrophils [39]. Bacterial single-cell sequencing showed that in addition to *gapA,* the two samples contained 12 intact genes encoding virulence factors derived from *S. pneumoniae* (Fig. 4). *SPH0456*+ (*cpsB*), *cps4J*, *cps4K*, and *cps4L* are pneumococcal polysaccharide capsule synthesis genes; *lmb* and *pavA* encode pneumococcal adhesins; *piuA* and *psaA_1* encode pneumococcal transporters; *nanB* encodes sialidase; *slrA* encodes peptidyl-prolyl *cis*-trans isomerase; *srtA* encodes sortase A; and *tig_ropA_2* encodes a trigger factor. *STER1442*, *STER1444*+ , *epsB*+ , and *tig_ropA_5* were isolated from *Streptococcus thermophilus*; *fbpA_6*, *psaA_3*, and *rfbB_1*+ from *Streptococcus gordonii*; and *ctrC_1* and *lbpA* from *Neisseria.* Concerning pneumococcal virulence factors, genes encoding pneumococcal cell surface proteins such as choline-binding proteins (*cbpD*, *cbpG*, *lytA*, and *lytB*) and cell wall anchoring proteins (*iga*, *nanA*, *pavB*/*pfbB*, *pfbA*, *zmpB*, and *zmpC*) were also detected in fragments. We previously reported that the orthologs of *iga*, *nanA*, *pfbA*, *zmpB*, and *zmpC* are distributed among closely related streptococcal species, including oral *Streptococcus*, which is consistent with our results [[40], [41], [42]].

Although the host species of several of the detected pneumococcal virulence factors remained unclear, several pneumococcal virulence factors were harbored by oral streptococci. *Neisseria ctrC* was detected in a single-cell isolate, OSU002-0007, which was predicted to be *Neisseria mucosa.* Another single-cell isolate containing *Neisseria lbpA* was not identified in this species. Bacterial single-cell sequencing allowed us to elucidate the level at which bacteria have specific genes.

In the MiGA ANI analysis, 10 of the 88 SAGs met >95 % of the criteria for species identification (Table 2). Although several genomes were predicted to be *S. pneumoniae* through GTDBtk taxonomy analysis (Table S3), MiGA ANI analysis showed that *S. pneumoniae* had the highest ANI value (91.4 %) in only one genome (OSU002-0038; Table 2). Although CheckM results indicated a high genomic completeness of 99 %, the ANI value was less than 95 %, indicating that no SAGs were virtually identified as *S. pneumoniae* in the samples. *S. pneumoniae* belongs to the mitis group of oral *Streptococcus* and cannot be distinguished from *S. mitis* or *S. oralis* through 16S rRNA sequencing, and some strains are difficult to identify even by biochemical tests [32,43,44]. Although the GTDBtk analysis identified 43 SAGs to the species level, some SAGs were misidentified as *S. pneumoniae,* indicating the difficulty in distinguishing bacteria from closely related species at the species level. ANI analysis also suggested five single-cell-isolated bacteria as potential novel species (Table 2).

**Table 2:** ANI analysis of single-cell-isolated genomes using the MiGA database.

Sample ID	Taxonomy defined by ANI analysis	The closest relatives found by MiGA in the database		Taxonomic novelty P-values
OSU001-0001	–	–	–	–
OSU001-0002	domain Bacteria	*Staphylococcus schleiferi* NZ AP014944 (38.19 % AAI)	*Staphylococcus schleiferi* NZ CP009470 (38.18 % AAI)	root 0.703, domain 0.682, phylum 0.0861, class 0.00459, order 0.00221, family 0.00356, genus 0.00644, species 0, subspecies 0, dataset 0.
OSU001-0003	–	–	–	–
OSU001-0004	genus *Streptococcus*	*Streptococcus oralis* subsp. tigurinus NZ AP018338 (90.67 % ANI)		root 1, domain 1, phylum 0.999, class 0.997, order 0.99, family 0.977, genus 0.939, species 0.00897, subspecies 0.000182, dataset 0.
OSU001-0005	–	–	–	–
OSU001-0006	–	–	–	–
OSU001-0007	–	–	–	–
OSU001-0008	–	–	–	–
OSU001-0009	–	–	–	–
OSU001-0010	–	–	–	–
OSU001-0011	family Fusobacteriaceae	*Fusobacterium periodonticum* NZ CP028108 (79.15 % AAI)	*Fusobacterium pseudoperiodonticum* NZ CP024704 (79.07 % AAI)	root 0.999, domain 0.999, phylum 0.996, class 0.992, order 0.973, family 0.937, genus 0.864, species 0.00269, subspecies 0.000182, dataset 0.
OSU001-0012	–	–	–	–
OSU001-0013	*Streptococcus salivarius*	*Streptococcus salivarius* NZ LS483366 (97.81 % ANI)	*Streptococcus salivarius* NZ CP015283 (97.7 % ANI)	root 1, domain 1, phylum 0.999, class 0.998, order 0.993, family 0.984, genus 0.958, species 0.0681, subspecies 0.00745, dataset 0.000206.
OSU001-0014	–	–	–	–
OSU001-0015	–	–	–	–
OSU001-0016	genus *Streptococcus*	*Streptococcus gordonii* str. Challis substr CH1 NC 009785 (89.37 % ANI)	*Streptococcus gordonii* NZ LS483341^T^ (89.36 % ANI)	root 1, domain 1, phylum 0.999, class 0.997, order 0.989, family 0.975, genus 0.933, species 0.00554, subspecies 0.000182, dataset 0.
OSU001-0017	*Rothia dentocariosa*	*Rothia dentocariosa* NZ CP054018 (97.14 % ANI)	*Rothia dentocariosa* ATCC 17931 NC 014643^T^ (96.7 % ANI)	root 1, domain 1, phylum 0.999, class 0.998, order 0.993, family 0.984, genus 0.957, species 0.0541, subspecies 0.00472, dataset 0.000206
OSU001-0018	–	–	–	–
OSU001-0019	–	–	–	–
OSU001-0020	–	–	–	–
OSU001-0021	class Bacilli	*Streptococcus mitis* NCTC 12261 NZ CP028414^T^ (45.8 % AAI)	*Streptococcus mitis* NZ CP046335 (45.8 % AAI)	root 0.927, domain 0.922, phylum 0.705, class 0.371, order 0.103, family 0.0189, genus 0.0202, species 0.000171, subspecies 0, dataset 0
OSU001-0022	–	–	–	–
OSU001-0023	–	–	–	–
OSU001-0024	genus *Streptococcus*	*Streptococcus gwangjuense* NZ CP032621^T^ (93.09 % ANI)		root 1, domain 1, phylum 0.999, class 0.998, order 0.992, family 0.981, genus 0.948, species 0.0137, subspecies 0.000363, dataset 0
OSU001-0025	genus *Streptococcus*	*Streptococcus oralis* NZ CP019562 (94.39 % ANI)		root 1, domain 1, phylum 0.999, class 0.998, order 0.992, family 0.982, genus 0.951, species 0.017, subspecies 0.000363, dataset 0
OSU001-0026	–	–	–	–
OSU001-0027	genus *Streptococcus*	Streptococcus oralis NZ CP065707 (91.47 % ANI)		root 1, domain 1, phylum 0.999, class 0.997, order 0.991, family 0.978, genus 0.942, species 0.0101, subspecies 0.000182, dataset 0
OSU001-0028	*Fusobacterium* sp. oral taxon 203	*Fusobacterium* sp. oral taxon 203 NZ CP016200 (97.91 % ANI)	*Fusobacterium nucleatum* subsp. nucleatum ATCC 23726 NZ CP028109 (94.94 % ANI)	root 1, domain 1, phylum 0.999, class 0.998, order 0.993, family 0.984, genus 0.958, species 0.0691, subspecies 0.00745, dataset 0.000206
OSU001-0029	–	–	–	–
OSU001-0030	–	–	–	–
OSU001-0031	–	–	–	–
OSU001-0032	class Clostridia	*Aminipila* sp. JN 18 NZ CP035281 (53.34 % AAI)	*Aminipila butyrica* NZ CP048649T (53.29 % AAI)	root 0.983, domain 0.982, phylum 0.932, class 0.852, order 0.595, family 0.267, genus 0.178, species 0.000343, subspecies 0, dataset 0.
OSU001-0033	–	–	–	–
OSU001-0034	–	–	–	–
OSU001-0035	–	–	–	–
OSU001-0036	genus *Streptococcus*	*Streptococcus gordonii* str. Challis substr CH1 NC 009785 (89.03 % ANI)	*Streptococcus gordonii* NZ CP046328 (89.02 % ANI)	root 1, domain 1, phylum 0.999, class 0.997, order 0.989, family 0.974, genus 0.931, species 0.00526, subspecies 0.000182, dataset 0.
OSU001-0037	–	–	–	–
OSU001-0038	family Leptotrichiaceae	*Pseudoleptotrichia goodfellowii* NZ AP019822T (72.87 % AAI)	*Leptotrichia* sp. oral taxon 212 NZ CP012410 (64.59 % AAI)	root 0.998, domain 0.998, phylum 0.994, class 0.987, order 0.953, family 0.891, genus 0.766, species 0.00252, subspecies 0.000182, dataset 0.
OSU001-0039	–	–	–	–
OSU001-0040	–	–	–	–
OSU001-0041	genus *Streptococcus*	*Streptococcus mitis* NCTC 12261 NZ CP028414T (93.97 % ANI)	*Streptococcus mitis* NZ CP046335 (93.95 % ANI).	root 1, domain 1, phylum 0.999, class 0.998, order 0.992, family 0.981, genus 0.951, species 0.0152, subspecies 0.000363, dataset 0.
OSU001-0042	–	–	–	–
OSU001-0043	family Fusobacteriaceae	*Fusobacterium nucleatum* subsp. vincentii 3 1 27 NZ CP007064 (69.85 % AAI)	*Fusobacterium nucleatum* subsp. vincentii 3 1 36A2 NC 022196 (69.85 % AAI)	root 0.998, domain 0.998, phylum 0.993, class 0.985, order 0.948, family 0.88, genus 0.75, species 0.00252, subspecies 0.000182, dataset 0.
OSU001-0044	phyum Firmicutes	*Pseudoclostridium thermosuccinogenes* NZ CP021850T (44.47 % AAI)	*Clostridium* sp. BNL1100 NC 016791 (44.28 % AAI)	root 0.9, domain 0.893, phylum 0.598, class 0.19, order 0.0615, family 0.0131, genus 0.0174, species 0.000114, subspecies 0, dataset 0
OSU001-0045	–	–	–	–
OSU001-0046	order Bacteroidales	*Prevotella denticola* NZ CP032056 (62.04 % AAI)	*Prevotella denticola* F0289 NC 015311 (62.03 % AAI)	root 0.995, domain 0.995, phylum 0.98, class 0.955, order 0.843, family 0.707, genus 0.429, species 0.00131, subspecies 0, dataset 0
OSU001-0047	–	–	–	–
OSU001-0048	–	–	–	–
OSU002-0001	genus *Streptococcus*	*Streptococcus* sp. oral taxon 061 NZ CP058258 (92.51 % ANI)	*Streptococcus* sp. oral taxon 431 NZ CP014264 (88.78 % ANI).	root 1, domain 1, phylum 0.999, class 0.998, order 0.991, family 0.98, genus 0.947, species 0.0112, subspecies 0.000363, dataset 0.
OSU002-0002	–	–	–	–
OSU002-0003	genus *Streptococcus*	*Streptococcus mitis* NZ CP014326 (93.7 % ANI)	*Streptococcus mitis* NZ CP028415 (92.84 % ANI)	root 1, domain 1, phylum 0.999, class 0.998, order 0.992, family 0.981, genus 0.95, species 0.0151, subspecies 0.000363, dataset 0
OSU002-0004	genus *Streptococcus*	*Streptococcus mitis* NZ CP046335 (93.07 % ANI)	*Streptococcus mitis* NCTC 12261 NZ CP028414T (93.02 % ANI)	root 1, domain 1, phylum 0.999, class 0.998, order 0.992, family 0.981, genus 0.948, species 0.0137, subspecies 0.000363, dataset 0
OSU002-0005	*Streptococcus* sp. FDAARGOS 192	*Streptococcus* sp. FDAARGOS 192 NZ CP020431 (96.78 % ANI)	*Streptococcus salivarius* JIM8777 NC 017595 (96.66 % ANI)	root 1, domain 1, phylum 0.999, class 0.998, order 0.993, family 0.983, genus 0.956, species 0.0456, subspecies 0.00164, dataset 0.000206
OSU002-0006	family Prevotellaceae	*Prevotella intermedia* NZ CP024727 (74.9 % AAI)	*Prevotella intermedia* NZ CP024732 (74.89 % AAI)	root 0.999, domain 0.999, phylum 0.995, class 0.988, order 0.959, family 0.903, genus 0.794, species 0.00252, subspecies 0.000182, dataset 0
OSU002-0007	genus *Neisseria*	*Neisseria mucosa* NZ CP053939 (96.22 % ANI)	*Neisseria subflava* NZ CP031251 (94.94 % ANI)	root 1, domain 1, phylum 0.999, class 0.998, order 0.993, family 0.983, genus 0.955, species 0.0351, subspecies 0.000727, dataset 0.000206
OSU002-0008	class Clostridia	*Pseudoclostridium thermosuccinogenes* NZ CP021850T (45.17 % AAI)	*Clostridium* sp. BNL1100 NC 016791 (45.06 % AAI)	
OSU002-0009	genus *Streptococcus*	*Streptococcus* sp. oral taxon 061 NZ CP058258 (92.4 % ANI)	*Streptococcus* sp. oral taxon 431 NZ CP014264 (89.1 % ANI)	root 1, domain 1, phylum 0.999, class 0.998, order 0.991, family 0.98, genus 0.946, species 0.011, subspecies 0.000363, dataset 0
OSU002-0010	–	–	–	–
OSU002-0011	–	–	–	
OSU002-0012	domain Bacteria	*Streptococcus ratti* NZ CP043405 (38.16 % AAI)	*Streptococcus mutans* NZ CP050270 (38.14 % AAI)	root 0.703, domain 0.682, phylum 0.0861, class 0.00459, order 0.00221, family 0.00356, genus 0.00644, species 0, subspecies 0, dataset 0
OSU002-0013	–	–	–	–
OSU002-0014	genus *Streptococcus*	*Streptococcus* sp. oral taxon 431 NZ CP014264 (93.19 % ANI)	*Streptococcus* sp. oral taxon 061 NZ CP058258 (88.34 % ANI)	root 1, domain 1, phylum 0.999, class 0.998, order 0.992, family 0.981, genus 0.949, species 0.0139, subspecies 0.000363, dataset 0
OSU002-0015	genus *Prevotella*	*Prevotella jejuni* NZ CP023863 (96.47 % ANI)	*Prevotella melaninogenica* NZ CP054010 (86.56 % ANI)	root 1, domain 1, phylum 0.999, class 0.998, order 0.993, family 0.983, genus 0.955, species 0.0407, subspecies 0.000908, dataset 0.000206
OSU002-0016	class Clostridia	*Herbinix luporum* NZ LN879430T (51.53 % AAI)	*Clostridium scindens* ATCC 35704 NZ CP036170T (51.42 % AAI)	root 0.978, domain 0.977, phylum 0.911, class 0.807, order 0.526, family 0.195, genus 0.132, species 0.000343, subspecies 0, dataset 0
OSU002-0017	genus *Leptotrichia*	*Leptotrichia* sp. oral taxon 212 NZ CP012410 (88.95 % ANI)		root 1, domain 1, phylum 0.999, class 0.997, order 0.989, family 0.974, genus 0.931, species 0.00526, subspecies 0.000182, dataset 0
OSU002-0018	–	–	–	–
OSU002-0019	–	–	–	–
OSU002-0020	genus Leptotrichia	*Leptotrichia* sp. oral taxon 212 NZ CP012410 (89.11 % ANI)		root 1, domain 1, phylum 0.999, class 0.997, order 0.989, family 0.974, genus 0.932, species 0.00532, subspecies 0.000182, dataset 0
OSU002-0021	genus *Streptococcus*	*Streptococcus gordonii* NZ CP017295 (89.2 % ANI)	*Streptococcus gordonii* str. Challis substr CH1 NC 009785 (89.2 % ANI)	root 1, domain 1, phylum 0.999, class 0.997, order 0.989, family 0.974, genus 0.932, species 0.00532, subspecies 0.000182, dataset 0
OSU002-0022	genus *Streptococcus*	*Streptococcus* sp. oral taxon 431 NZ CP014264 (93.25 % ANI)	*Streptococcus* sp. oral taxon 061 NZ CP058258 (88.66 % ANI)	root 1, domain 1, phylum 0.999, class 0.998, order 0.992, family 0.981, genus 0.949, species 0.0143, subspecies 0.000363, dataset 0
OSU002-0023	*Gemella sanguinis*	*Gemella sanguinis* NZ CP046313 (97.46 % ANI)		root 1, domain 1, phylum 0.999, class 0.998, order 0.993, family 0.984, genus 0.957, species 0.0608, subspecies 0.006, dataset 0.000206.
OSU002-0024	genus *Streptococcus*	*Streptococcus mitis* NZ CP014326 (93.53 % ANI)	*Streptococcus mitis* NZ CP046335 (92.82 % ANI)	root 1, domain 1, phylum 0.999, class 0.998, order 0.992, family 0.981, genus 0.95, species 0.0149, subspecies 0.000363, dataset 0
OSU002-0025	genus *Gemella*	*Gemella morbillorum* NZ CP046314 (96.54 % ANI)	*Gemella morbillorum* NZ LS483440T (96.52 % ANI)	root 1, domain 1, phylum 0.999, class 0.998, order 0.993, family 0.983, genus 0.956, species 0.0421, subspecies 0.00109, dataset 0.000206
OSU002-0026	genus *Streptococcus*	*Streptococcus* sp. oral taxon 431 NZ CP014264 (93.14 % ANI)	*Streptococcus* sp. oral taxon 061 NZ CP058258 (88.64 % ANI)	root 1, domain 1, phylum 0.999, class 0.998, order 0.992, family 0.981, genus 0.949, species 0.0139, subspecies 0.000363, dataset 0
OSU002-0027	–	–	–	–
OSU002-0028	–	–	–	–
OSU002-0029	class Bacilli	*Bacillus infantis* NRRL B 14,911 NC 022524 (47.87 % AAI)	*Bacillus marisflavi* NZ CP033051 (47.66 % AAI)	root 0.953, domain 0.95, phylum 0.81, class 0.587, order 0.233, family 0.0639, genus 0.0446, species 0.000171, subspecies 0, dataset 0
OSU002-0030	–	–	–	–
OSU002-0031	*Gemella morbillorum*	*Gemella morbillorum* NZ CP046314 (96.87 % ANI)	*Gemella morbillorum* NZ LS483440T (96.71 % ANI)	root 1, domain 1, phylum 0.999, class 0.998, order 0.993, family 0.984, genus 0.956, species 0.0477, subspecies 0.00182, dataset 0.000206
OSU002-0032	genus *Streptococcus*	*Streptococcus* sp. oral taxon 061 NZ CP058258 (92.45 % ANI)	*Streptococcus* sp. oral taxon 431 NZ CP014264 (89.1 % ANI)	root 1, domain 1, phylum 0.999, class 0.998, order 0.991, family 0.98, genus 0.946, species 0.011, subspecies 0.000363, dataset 0
OSU002-0033	*Rothia dentocariosa*	*Rothia dentocariosa* NZ CP054018 (97.88 % ANI)	*Rothia dentocariosa* ATCC 17931 NC 014643T (96.7 % ANI)	root 1, domain 1, phylum 0.999, class 0.998, order 0.993, family 0.984, genus 0.958, species 0.0681, subspecies 0.00745, dataset 0.000206
OSU002-0034	class Mollicutes	*Mycoplasma arthritidis* NZ LR215047T (54.0 % AAI)	*Mycoplasma arthritidis* 158L3 1 NC 011025 (53.94 % AAI)	root 0.985, domain 0.984, phylum 0.938, class 0.866, order 0.622, family 0.304, genus 0.195, species 0.000343, subspecies 0, dataset 0.
OSU002-0035	genus *Gemella*	*Gemella haemolysans* NZ LR134484 (91.53 % ANI)	*Gemella haemolysans* NZ CP050965 (87.67 % ANI)	root 1, domain 1, phylum 0.999, class 0.997, order 0.991, family 0.978, genus 0.943, species 0.0101, subspecies 0.000182, dataset 0
OSU002-0036	genus *Rothia*	*Rothia dentocariosa* NZ CP054018 (91.73 % ANI)	*Rothia dentocariosa* ATCC 17931 NC 014643T (91.26 % ANI)	root 1, domain 1, phylum 0.999, class 0.997, order 0.991, family 0.979, genus 0.944, species 0.0103, subspecies 0.000182, dataset 0
OSU002-0037	genus *Streptococcus*	*Streptococcus mitis* NZ CP046335 (93.07 % ANI)	*Streptococcus mitis* NCTC 12261 NZ CP028414T (93.06 % ANI)	root 1, domain 1, phylum 0.999, class 0.998, order 0.992, family 0.981, genus 0.948, species 0.0137, subspecies 0.000363, dataset 0
OSU002-0038	genus *Streptococcus*	*Streptococcus pneumoniae* A026 NC 022655 (91.81 % ANI)	*Streptococcus pneumoniae* NZ CP028436 (91.73 % ANI)	root 1, domain 1, phylum 0.999, class 0.997, order 0.991, family 0.979, genus 0.944, species 0.0104, subspecies 0.000182, dataset 0
OSU002-0039	genus *Streptococcus*	*Streptococcus gwangjuense* NZ CP032621T (93.14 % ANI)		root 1, domain 1, phylum 0.999, class 0.998, order 0.992, family 0.981, genus 0.949, species 0.0139, subspecies 0.000363, dataset 0
OSU002-0040	–	–	–	–
OSU002-0041	genus *Leptotrichia*	*Leptotrichia* sp. oral taxon 212 NZ CP012410 (89.24 % ANI)		root 1, domain 1, phylum 0.999, class 0.997, order 0.989, family 0.974, genus 0.932, species 0.00543, subspecies 0.000182, dataset 0
OSU002-0042	–	–	–	–
OSU002-0043	genus *Streptococcus*	*Streptococcus* sp. oral taxon 431 NZ CP014264 (91.33 % ANI)	*Streptococcus* sp. oral taxon 061 NZ CP058258 (88.29 % ANI)	root 1, domain 1, phylum 0.999, class 0.997, order 0.991, family 0.978, genus 0.942, species 0.0101, subspecies 0.000182, dataset 0
OSU002-0044	phylum Firmicutes	*Abiotrophia defectiva* NZ CP053988T (40.73 % AAI)	*Enterococcus durans* NZ LR607335 (40.6 % AAI)	root 0.846, domain 0.835, phylum 0.38, class 0.0131, order 0.00638, family 0.0045, genus 0.00796, species 0, subspecies 0, dataset 0
OSU002-0045	genus *Streptococcus*	*Streptococcus* sp. oral taxon 431 NZ CP014264 (93.19 % ANI)	*Streptococcus* sp. oral taxon 061 NZ CP058258 (88.28 % ANI)	root 1, domain 1, phylum 0.999, class 0.998, order 0.992, family 0.981, genus 0.949, species 0.0139, subspecies 0.000363, dataset 0
OSU002-0046	–	–	–	–
OSU002-0047	class Bacilli	*Carnobacterium divergens* NZ LT992558 (55.91 % AAI)	*Carnobacterium divergens* NZ CP016843 (55.52 % AAI)	root 0.989, domain 0.989, phylum 0.957, class 0.908, order 0.694, family 0.424, genus 0.231, species 0.0004, subspecies 0, dataset 0
OSU002-0048	genus *Streptococcus*	*Streptococcus mitis* NZ CP014326 (93.76 % ANI)	*Streptococcus mitis* NZ CP046335 (92.98 % ANI)	root 1, domain 1, phylum 0.999, class 0.998, order 0.992, family 0.981, genus 0.95, species 0.0151, subspecies 0.000363, dataset 0

## 4 Discussion

Saliva plays a multifaceted role in digestion, tooth remineralization, and oral cavity cleaning. However, it is also a vector of droplet infections. Daily activities such as talking, coughing, and sneezing produce large amounts of respiratory droplets that are subsequently deposited on dry surfaces [45]. These droplets facilitate the transmission of bacteria such as *S. pneumoniae* [46]. However, many microorganisms in the saliva are unidentified. Identifying the salivary microbiome is crucial to understand the mode of human-to-human transmission of microorganisms and/or AMR genes. This study would contribute to expanding oral microbiome data resources.

*S. pneumoniae* can acquire genetic material from oral streptococcal species, resulting in drug resistance and serotype replacement [[4], [5], [6], [7]]. Jensen et al. comprehensively assessed the involvement of homologous recombination between oral streptococci and *S. pneumoniae* in acquiring β-lactam drug resistance. This study focused on the diversity at the DNA and amino acid levels of the transpeptidase region of *pbp2x* in 107 strains, *pbp2b* in 96 strains, and *pbp1a* in 88 strains of oral *Streptococcus* [4]. The findings revealed that polymorphic sites arising from spontaneous mutations in *pbp* accounted for 39 % of all polymorphic sites observed in susceptible and resistant strains of *S. mitis*, *S. oralis*, and *Streptococcus infantis*. By contrast, extensive sequence variation was observed exclusively in resistant strains of *S. pneumoniae*. These results suggested that the previously diversified sequence in oral streptococci was imported by *S. pneumoniae* possibly because of the selective pressure exerted by antimicrobial agents.

In 2020, Ganaie et al. reported the discovery of the 100th pneumococcal capsular serotype, designated as 10D [7]. This study revealed that the capsular synthesis genes of serotype 10D exhibited three large regions of homology with genes arranged in the same order (syntenic) as those found in serotypes 6C and 39 and the capsular synthesis genes of *S. mitis* SK145. Notably, the syntenic region of 10D with SK145 spanned approximately 6000 bp and included a short fragment of *wciNα* at the 5ʹ end. The presence of this nonfunctional *wciNα* fragment provided compelling evidence of interspecies gene transfer from oral streptococci to *S. pneumoniae*. Moreover, the sequence of *wcrO*_10D_, a capsular synthesis gene cluster of serotype 10D, displayed low homology (40–50 % amino acid identity) with the *wcrO* genes of serotypes 33C, 34, 35F, and 36 despite the sequencing of the capsular synthesis gene cluster in over 20,000 pneumococcal strains. By contrast, *wcrO*_10D_ exhibited surprisingly high homology (94 % amino acid identity) with *RS00925* from *S. mitis* SK145. These findings suggest that the 100th capsular serotype 10D arose from the acquisition of the *S. mitis* gene by *S. pneumoniae*. Resistance and diversification of capsular types of *S. pneumoniae* pose significant threats to human health, and understanding the sources of this diversity is crucial. These results indicate that oral streptococci serve as an external genetic pool for pneumococci. In the present study, we detected the virulence factors of *S. thermophilus*, *S. gordonii*, and genus *Neisseria*, in addition to pneumococcal virulence factors. Commensal and pathogenic bacteria may share genes across a wider range of species than has been reported. Thus, elucidating the genetic diversity of the oral microbiome is important.

In the present study, saliva samples from the same individuals were classified as inactivated or viable and then analyzed using 16S rRNA sequencing, metagenomic shotgun sequencing, and bacterial single-cell sequencing. Both inactivated and viable samples were suitable for analysis, but the inactivated samples were preferred when dealing with samples that may pose a risk to analysts, such as those from COVID-19-infected individuals. Genomic sequencing facilitated the exploration of the metabolic systems of unculturable bacteria present in the samples, potentially allowing the cultivation of previously unculturable bacteria from viable samples.

Traditionally, metagenomic shotgun sequencing has been used to study gene functions in the microbiome. However, metagenome shotgun analysis involves binning to identify bacterial species and then constructing a metagenome-assembled genome to search for gene distribution [47]. Although the binning technology has improved and contributed to the identification of many bacteria, the high homology of essential genes among species makes complete classification extremely difficult. In other words, identifying which bacteria possess the genes detected by metagenome shotgun sequencing is a future challenge and is expected to be improved by short- and long-read hybrid analysis, long-read deep sequencing combined with Hi-C-seq, and other techniques [48,49].

Using OMNI stocks, a recent study has obtained 742 oral bacterial SAGs from two healthy volunteers, of which 450 SAGs consisting of 27 genera have remained after excluding SAGs with 10 % contamination rate or 0 % completeness [50]. The top 5 most abundant genera in this study were *Streptococcus* (60.67 %), *Prevotella* (9.78 %), *Gemella* (7.56 %), F0040 (*Alloprevotella*; 6.89 %), and *Granulicatella* (3.11 %) based on GTDB classification. In the present study, the top 5 most abundant genera were *Streptococcus* (38.64 %), *Prevotella* (10.23 %), *Gemella* (7.95 %), TM7x (7.95 %), and *Fusobacterium* (5.68 %). The differences in genera and their proportions indicate the diverse composition of the oral microbiome among individuals. In addition, while our 16S rRNA sequencing detected 60 genera, 27 genera were detected from the 450 SAGs. This result suggests that 16S rRNA sequencing remains a powerful tool for investigating the composition of the oral microbiome.

The single-cell analysis performed in this study proved to be highly effective, allowing the precise identification of genes present in individual bacteria. High genome integrity is obtained by ensuring that each bacterial cell is intact and the genome is not damaged; OMNI and glycerol stocks maintain the intactness of oral bacteria. In addition, the use of preisolated bacteria eliminates the need for binning, which poses challenges in terms of improving accuracy. Sequencing DNA in a single bacterial cell can reveal the host of mobile genetic elements, such as plasmids and phages, whereas Hi-C-seq provides a community composition profile. Elucidating the dynamics of gene transfer between bacterial species at high frequencies is essential to understand the spread of resistance genes through saliva and develop effective control strategies. Future studies could combine multiple methods to elucidate the human microbiota structure and identify previously unrecognized bacterial species and genetic characteristics.

## Ethics approval and consent to participate

The study was conducted with written informed consent from the donor and approved by the Institutional Review Board of Osaka University Graduate School of Dentistry (R4-E4).

## Consent for publication

Not applicable.

## Availability of data and materials

The 16S rRNA amplicon sequencing, metagenome-shotgun sequencing, and bacterial single-cell sequencing data are deposited in the DNA Data Bank of Japan under BioProject PRJDB16375. The DRR run numbers are DRR502934-DRR503025.

## Funding

This study was partly supported by 10.13039/100009619AMED (JP20wm0325001), the 10.13039/501100001691Japan Society for the Promotion of Science KAKENHI (grant numbers 20KK0210, 21K10137, 22H03262, 22K19618, 22K19619, and 23H03073), 10.13039/100007449Takeda Science Foundation, the Joint Research Program of the Research Center for GLOBAL and LOCAL Infectious Diseases, 10.13039/501100007510Oita University (2022B05), and the Drug Discovery Science Division, Open and Transdisciplinary Research Initiatives, 10.13039/501100004206Osaka University. This work was conducted as part of “The Nippon Foundation - Osaka University Project for Infectious Disease Prevention.” The funders had no role in the study design, data collection or analysis, decision to publish, or preparation of the manuscript.

## CRediT authorship contribution statement

**Masaya Yamaguchi:** Writing – review & editing, Writing – original draft, Visualization, Validation, Resources, Project administration, Methodology, Investigation, Funding acquisition, Formal analysis, Data curation, Conceptualization. **Toshihiro Uchihashi:** Writing – review & editing, Funding acquisition, Data curation. **Shigetada Kawabata:** Writing – review & editing, Funding acquisition, Conceptualization.

## Declaration of competing interest

The authors declare that they have no competing interests.

## Abbreviations

(AMR): antimicrobial resistance
(SAG): single-amplified genome
(MAGs): metagenome-assembled genomes
(ANI): average nucleotide identity
(CARD): Comprehensive Antibiotic Resistance Database
(VFDB): virulence factor database
