# Supplementary material for: Hybrid sequence-based analysis reveals the distribution of bacterial species and genes in the oral microbiome at a high resolution
Source: Biochem Biophys Rep. 2024 Apr 26;38:101717. doi: 10.1016/j.bbrep.2024.101717 (PMC11066573; doi:10.1016/j.bbrep.2024.101717)
Supplement: Multimedia component 1 [file mmc1.pdf]

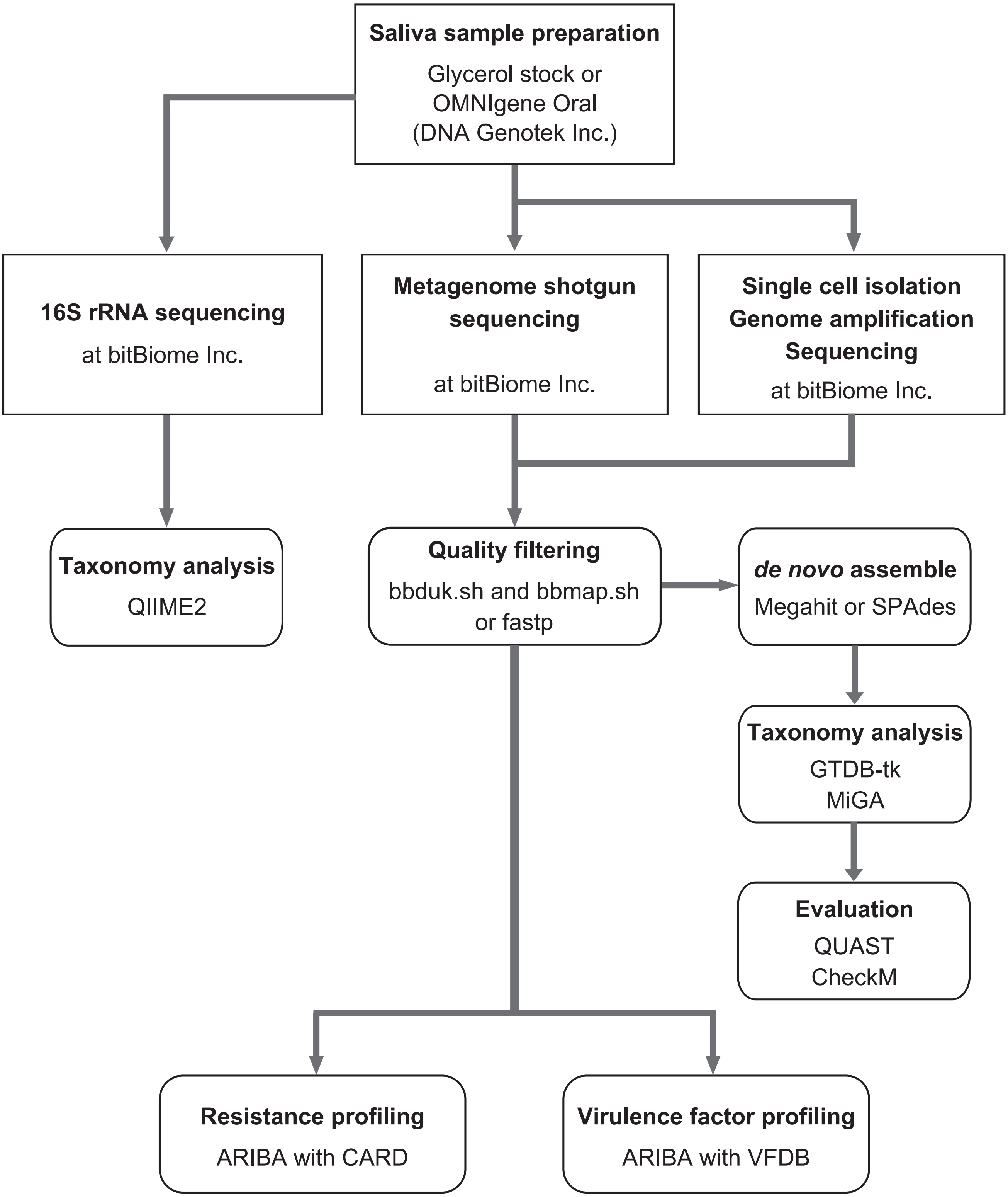

Supplementary Figure 1. Workflow of the metagenomic analysis for a saliva microbiome.

Rectangles indicate in vitro analysis, whereas rounded rectangles indicate in silico analysis.
